# Low dietary inclusion of nutraceuticals from microalgae improves feed efficiency and modifies intermediary metabolisms in gilthead sea bream (*Sparus aurata*)

**DOI:** 10.1038/s41598-020-75693-3

**Published:** 2020-10-29

**Authors:** Erick Perera, David Sánchez-Ruiz, María Isabel Sáez, Alba Galafat, André Barany, Miriam Fernández-Castro, Antonio Jesús Vizcaíno, Juan Fuentes, Tomás Francisco Martínez, Juan Miguel Mancera, Francisco Javier Alarcón, Juan Antonio Martos-Sitcha

**Affiliations:** 1 Department of Biology, Faculty of Marine and Environmental Sciences, Instituto Universitario de Investigación Marina (INMAR), Campus de Excelencia Internacional del Mar (CEI·MAR), University of Cádiz, 11519 Puerto Real, Cádiz Spain; 2 Department of Biology and Geology, Campus de Excelencia Internacional del Mar (CEI·MAR), University of Almería, 04120 Almería, Spain; 3 Centre of Marine Sciences (CCMar), Universidade do Algarve, Faro, Portugal; 4 Present Address: Futuna Blue España S.L., Dársena Comercial Pesquera s/n, 11500 El Puerto de Santa María, Cádiz Spain

**Keywords:** Physiology, Animal physiology, Ichthyology, Biochemistry, Enzymes, Nutrition

## Abstract

The aim of this work was to evaluate two functional feeds for the gilthead seabream, *Sparus aurata*, containing low inclusion of two microalgae-based products (LB-GREENboost, LB_Gb_; and LB-GUThealth, LB_Gh_). Fish (12–13 g) were fed for 13 weeks a control diet or one of the four diets supplemented with both products at 0.5% or 1%. LB_Gb_ and LB_Gh_ did not affect specific growth rate or survival, but increased feed efficiency by decreasing feed intake and enlarging the intestines. LB_Gb_ increased hepatosomatic index and reduced cortisol levels in plasma, while both products lowered plasma lactate. Extensive metabolite and metabolic enzyme profiling revealed that microalgae supplementations, especially 1% LB_Gh_: (i) decrease plasma lactate and increase hepatic glycogen, (ii) reduce hepatic gluconeogenesis, (iii) enhance hepatic lipogenic activity and lipid secretion, (iv) led fish to double triglyceride content in muscle and to stimulate its lipid oxidative capacity, and (v) increase the content of monounsaturated fatty acids and the omega-3 alpha-linolenic acid in muscle. This study demonstrates that both microalgae-based products are suited to improve feed efficiency and orchestrate significant changes in the intermediary metabolism in gilthead seabream juveniles.

## Introduction

World aquaculture production is continuously growing at a high rate, but the European industry is stagnant and needs to advance to a more competitive and high-performance industry, while ensuring high level of sustainability^1^. One major issue related with both economic and environmental sustainability is the replacement of fish meal (FM) and fish oil (FO) in aquafeeds. Significant achievements have been made during the last decade, and the fish aquaculture industry has significantly reduced the dependence on these marine ingredients by incorporating balanced mixtures of plant meals and oils in feed formulations^2–5^, and higher replacement rates are expected through the use of novel alternatives such as genetically engineered oilseed crops and the use of microalgae^6^.

Microalgae and microalgae-derived products are rich in proteins^7^ and lipids with high amount of highly unsaturated fatty acids^8^, thus they have been evaluated in different fish species as alternatives for FM and FO. Several studies indicated that microalgae inclusion in freshwater fish diets does not affect growth rate^9,10^, or can induce positive effects on their growth, metabolism, and meat quality^11^. Results are more variable in salmonids and marine fish, depending on the microalgae species employed and the level of inclusion in diet (e.g. salmonids^12–14^, gilthead sea bream^15–17^). Although the inclusion of high level of microalgae in most fish feeds is still limited by high production costs, different companies have announced algae-based products to be used in high-valued commercial fish (e.g. salmonids) as alternatives to FO^6^, and different products (e.g. AlgaPrimeDHA) are already being produced at large-scale through fermentation-based technologies^18^.

While the use of microalgae-derived products as alternative raw material in fish feeds has received most of the attention, there is also an increasing interest in their use as functional feed additives. In this sense, the use of microalgae products at relatively low level (< 10%) in fish functional feeds has the potential to improve aquaculture production by enhancing growth and/or feed efficiency^19–21^, two features that have been traditionally improved through the optimization of feed formulations and feeding practices. Feed efficiency, in particular, is difficult to increase using phenotypic trait selection. Other aspects that can be improved by the use of functional feeds are wellbeing, health, stress resistance, or product quality, which are also of a great interest today for both producers and consumers. Functional feeds (or nutraceutical components in feed) trigger, by definition, beneficial effects upon physiological functions^22^. Bioactive compounds from microalgae such as protein, polyunsaturated fatty acids, polysaccharides, carotenoids, vitamins and minerals, phenolic compounds, volatile compounds, and sterols play important roles in functional feeds for both humans and livestock^20,23,24^. The most widely microalgae-derived product used by the aquaculture industry is astaxanthin, mainly to improve the coloration, but also other important physiological processes^25^. However, different microalgae have immunostimulating and health promoting effects properties in fish^26^, or improve growth and fillet quality^27^. For instance, the addition of moderate levels (~ 10%) of a *Chlorella*-derived product in feed is able to ameliorate plat-induced enteritis in salmonids^28^, and ~ 4.5–5.0% inclusion of *Isochrysis galbana* biomass in diets for the marine fish *Trachinotus ovatus*, improves growth performance, lipid deposition and content of muscular n-3 fatty acids, DHA, and EPA^27^.Yet, studies on microalgae-derived products used at lower inclusion levels in diets for farmed fish are scarce, despite this would have a positive impact on feed cost. In other livestock such as broiler chicks, feed supplementation with only 0.1–0.2% of microalgae products, considered as a very-low inclusion level, produces positive effects such as the improvement of fatty acid composition of meat without affecting the growth performance^29^.

The aim of this work was to evaluate the potential benefits of two functional feeds for the gilthead seabream, which contain low level of two microalgae-based products, LB-GREENboost (LB_Gb_) and LB-GUThealth (LB_Gh_), developed by *LifeBioencapsulation S.L.*, a spin-off from the University of Almería (Spain). The gilthead seabream is one of the main fish species farmed in Europe, especially in the Mediterranean region. In particular, the effects of low dietary inclusion (0.5% and 1%) of these products on growth performance, nutrient utilization, and intermediary metabolism were studied after a 13-week feeding trial. This study shows that the products evaluated are suited for improving some important indicators of culture performance and physiological condition of gilthead seabream juveniles, thus revealing the potential for their inclusion in new functional feeds for this, or even others cultured species.

## Material and methods

### Ethics

Fish were kept and handled following the guidelines for experimental procedures in animal research of the Ethics and Animal Welfare Committee of the University of Cadiz, according to the Spanish (RD53/2013) and European Union (2010/63/UE) legislation. The Ethical Committee from the Autonomous Andalusian Government approved the experiments (Junta de Andalucía reference number 04/04/2019/056).

### Diets

Five isoproteic, isolipidic and isoenergetic diets were formulated with a composition that is close to that of commercial feeds for the gilthead seabream, and produced at the University of Almería facilities (Experimental feeds Service; https://www.ual.es/stecnicos_spe). FM and FO were included at 20% and 9.2%, respectively, in all experimental diets. This formulation constituted the control diet (CTRL). In addition, two commercial compounds extracted from microalgae, (i) LB-GREENboost (LB_Gb_) and (ii) LB-GUThealth (LB_Gh_) developed by *LifeBioencapsulation S.L.* (Almería, Spain), were added at 0.5% and at 1%, constituting the four supplemented diets (Table 1). LB_Gb_ (crude protein 57.0%, crude fat 6.4%, crude fibre 0.4%, crude ash 9.4%, and moisture 7.0%) and LB_Gh_ (crude protein 56.0%, crude fat 2.0%, crude fibre 0.2%, crude ash 12.7%, and moisture 8.8%) are concentrated mixtures containing 800 g kg^−1^ and 200 g kg^−1^, respectively, of a blend of microalgae extracts. The rest of compounds used in those commercial products are lipotropic substances (choline and betaine) and calcium carbonate used as excipients. The ingredient composition and fatty acid profile of experimental diets are shown in Tables 1 and 2, respectively.

**Table 1:** Ingredients and chemical composition of the experimental diets.

	CTRL	LB_Gb0.5_	LB _Gb1_	LB_Gh0.5_	LB_Gh1_
**Ingredient composition (g kg**^**−1**^**)**					
Fish meal LT-94^a^	200	200	200	200	200
Lysine^b^	12	12	12	12	12
Methionine^c^	5	5	5	5	5
LB-GREENboost (LB_Gb_)	–	5	10	–	–
LB-GUThealth (LB_Gh_)	–	–	–	5	10
Krill meal^d^	25	25	25	25	25
Gluten wheat^e^	130	130	130	130	130
Soybean protein concentrate^f^	342	342	342	342	342
Fish oil^g^	92	92	92	92	92
Soybean oil^h^	44	44	44	44	44
Soybean lecithin^i^	10	10	10	10	10
Wheat meal^j^	94	89	84	89	84
Betain^k^	5	5	5	5	5
Vitamin and mineral premix^l^	20	20	20	20	20
Vitamin C^m^	1	1	1	1	1
Guar gum^n^	10	10	10	10	10
Alginate^o^	10	10	10	10	10
**Proximate composition (g kg**^**−1**^**)**					
Moisture	90.3	89.7	90.5	89.6	88.9
Crude protein	462.1	458.7	459.4	460	463.6
Crude lipid	184.2	182.3	180.7	179.3	178.2
Ash	99.4	102.7	104.8	98.5	103.0
Nitrogen free extract	254.5	256.3	255.5	262.2	255.2

CTRL: control; LB_Gb0.5_: LB-GREENboost (0.5%); LB_Gb1_: LB-GREENboost (1%); LB_Gh0.5_: LB-GUThealth (0.5%); LB_Gh1_: LB-GUThealth (1%).

^a^69.4% crude protein, 12.3% crude lipid (Norsildemel, Bergen, Norway).

^b,c,h,k^Lorca Nutricion Animal S.A. (Murcia, Spain).

^d^45% crude protein, 9% crude lipid (Bacarel, UK).

^e^78% crude protein, 1% ash, 105 moisture (Lorca Nutricion Animal S.A., Murcia, Spain).

^f^65% crude protein, 8% crude lipid (DSM, France).

^g^AF117DHA (Afamsa, Spain).

^i^P700IP (Lecico, DE).

^j^Local provider (Almería, Spain).

^l^Provided by Lifebioencapsulation S.L.Vitamins (mg kg^−1^): vitamin A (retinyl acetate), 2,000,000 UI; vitamin D3 (DL-cholecalciferol), 200,000 UI; vitamin E (Lutavit E50), 10,000 mg; vitamin K3 (menadione sodium bisulfitete), 2500 mg; vitamina B1(thiamine hydrochloride), 3000 mg; vitamin B2 (riboflavin), 3000 mg; calcium pantothenate, 10,000 mg; nicotínico acid, 20,000 mg; vitamin B6 (pyridoxine hydrochloride), 2000 mg; vitamin B9 (folic acid), 1,500 mg; vitamin B12 (cyanocobalamin), 10 mg vitamin H (biotin), 300 mg; inositol, 50,000 mg; betaine (Betafin S1), 50,000 mg. Minerals (mg kg^−1^): Co (cobalt carbonate), 65 mg; Cu (cupric sulfate), 900 mg; Fe (iron sulFate), 600 mg; I (potassium iodide), 50 mg; Mn (manganese oxide), 960 mg; Se (sodium selenite), 1 mg; Zn (zinc sulphate) 750 mg; Ca (calcium carbonate), 18.6%; (186,000 mg); KCl, 2.41%; (24,100 mg); NaCl, 4.0% (40,000 mg).

^m,n,o^EPSA, Spain.

**Table 2:** Fatty acid content (g fatty acid kg^−1^) of the microalgae additives LB-GREENboost (*LB*_*Gb*_) and LB-GUThealth (*LB*_*Gh*_), and fatty acid profiles (% total fatty acids) of the experimental diets (% of total FAs).

	Microalgal products (g fatty acid kg^−1^)		Experimental diets (% total fatty acids)				
	LB_Gb_	LB_Gh_	CTRL	LB_Gb0.5_	LB_Gb1_	LB_Gh0.5_	LB_Gh1_
14:0	4.61	1.32	2.11	2.10	2.11	2.11	2.12
16:0	12.39	3.54	16.43	16.94	16.90	16.45	16.39
18:0	2.15	0.61	5.25	5.49	5.24	5,15	5,17
16:1n7	6.62	1.89	3.09	3.04	3.06	3.10	3.11
18:1n7	0.15	0.04	1.88	1.83	1,85	1.87	1.85
18:1n9	4.38	1.25	19,76	20.27	20.27	20.28	20.29
20:1n9	nd	nd	1.16	1.18	1.15	1.10	1.09
16:2n4	1.24	0.35	0.58	0.56	0.58	0.56	0.57
18:2n6	4.59	1.31	24.47	23.27	23.57	24.36	24.34
18:3n3	11.17	3.19	1.20	1.30	1.28	1.26	1.23
16:3n4	0.85	0.24	0.43	0.41	0.42	0.42	0.43
18:4n3	8.93	2.55	0.56	0.67	0.61	0.51	0.51
20:4n6	0.63	0.18	1.17	1.13	1.12	1.17	1.16
20:4n3	nd	nd	0.40	0.38	0.38	0.40	0.40
20:5n3, EPA	3.05	0.87	4.22	4.15	4.14	4.14	4.15
22:5n3	0.71	0.20	1.18	1.13	1.11	1.12	1.13
22:6n3, DHA	2.32	0.66	12.70	12.45	12.46	12.49	12.50
∑SFA			23.79	24.52	24.24	23.72	23.69
∑MUFA			25.88	26.33	26.34	26.35	26.33
∑PUFA			45.91	44.48	44.67	45.47	45.43
Other FA			3.42	3.70	3.75	3.48	3.56
∑n-3			20.26	20.08	19.98	19.93	19.93
∑n-6			25.65	24.40	24.69	25.53	25.50
∑n-9			20.91	21.45	21.42	21.38	21.38
n3/n6			0.79	0.82	0.81	0.78	0.78
EPA/DHA			0.33	0.33	0.33	0.33	0.33

*LB*_*Gb0.5*_: LB-GREENboost (0.5%); *LB*_*Gb1*_: LB-GREENboost (1.0%); *LB*_*Gh0.5*_: LB-GUThealth (0.5%); *LB*_*Gh1*_: LB-GUThealth (1.0%).

nd: notdetected.

### Feeding protocol and sampling procedures

After an initial acclimation period (10 days) to the experimental facility (CTAQUA, El Puerto de Santa María, Cádiz, Spain), gilthead sea bream juveniles with 12–13 g of initial mean body weight were randomly distributed in fifteen 100-L tanks (n = 30 fish per tank, 90 fish per experimental diet) coupled to a recirculation aquaculture system (RAS), equipped with physical and biological filters, and programmable temperature and O_2_ devices. Water temperature was set constant at 22 ± 0.5 ºC. Oxygen content of outlet water remained higher than 85% saturation, and day-length followed the natural changes at our latitude (36º35′06″ N; 06º13′48″ W). Experimental diets were offered to visual satiety three times per day and 6 days per week from February to May (13-week feeding trial). Fish were counted and group-weighed every 3 weeks, and feed intake was recorded for each experimental replicate to calculate growth performance parameters. No mortalities were registered in any experimental group.

At the end of the trial (day 87), overnight fasted fish (4 fish per tank, 12 per experimental condition) were randomly selected, deeply anaesthetised with clove oil, and then sampled for blood and tissue collection. Blood was drawn from caudal vessels with heparinised syringes, centrifuged at 3000×*g* for 20 min at 4 ºC, and plasma samples were snap-frozen in liquid nitrogen and stored at − 80 ºC until biochemical and hormonal analysis. Prior to tissue collection, fish were killed by cervical section, and livers were extracted and weighed. Intestine was taken for length measurements. Samples of liver and white skeletal muscle were rapidly taken, snap-frozen in liquid nitrogen, and stored at − 80 ºC until biochemical analyses.

### Growth performance and biometric parameters

The following growth parameters were evaluated: (i) specific growth rate (SGR) = (100 × (ln final body weight − ln initial body weight)/days; (ii) weight gain (WG) = (100 × (body weigh increase)/initial body weight; (iii) feed efficiency (FE) = weight gain/total feed intake; and (iv) condition factor = (100 × body weight)/fork length. Biometric indices were estimated in accordance with the following equations: (i) Hepatosomatic index (HSI) = (100 × liver weight)/fish weight; and (ii) Intestine length index (ILI) = (100 × L_i_)/L_b_, where L_i_ and L_b_ are the intestine and fork body length, respectively.

### Biochemical parameters of the plasma

Plasma cortisol levels were measured with a commercial Cortisol Enzyme Immunoassay Kit from ARBORASSAYS (NCAL International Standard Kit, DETECTX, K003). Glucose, lactate and triglycerides levels in plasma were measured using commercial kits from SPINREACT (St. Esteve de Bas, Girona, Spain) adapted to 96-well microplates. Plasma total protein concentration was determined with a BCA Protein AssayKit (PIERCE, Thermo Fisher Scientific, USA, #23225) using BSA as the standard. All assays were performed using a POWERWAVE 340 microplate spectrophotometer (Bio-Tek Instruments, Winooski, VT, USA) using the KCJUNIOR data analysis software for MICROSOFT.

### Biochemical parameters of the liver and muscle

Frozen tissues used for the assay of metabolites were homogenized by ultrasonic disruption in 7.5 volumes ice-cold 0.6 N perchloric acid, neutralized using 1 M KCO_3_, centrifuged (30 min, 3220×*g* and 4 ºC), and then supernatants isolated to determine tissue metabolites. Tissue triglycerides and lactate levels were determined spectrophotometrically with commercial kits (SPINREACT, see above). Tissue glycogen concentration was quantified using the method described from^30^, where glucose obtained after glycogen breakdown with amyloglucosidase (SIGMA-ALDRICH A7420) was determined with a commercial kit (SPINREACT) as described before.

### Activity of metabolic enzymes in liver and muscle

Frozen tissues for enzyme activity assays were homogenized by ultrasonic disruption in 10 volumes of ice-cold homogenization buffer (50 mM imidazole, 1 mM 2-mercaptoethanol, 50 mM NaF, 4 mM EDTA, 0.5 mM phenylmethylsulfonyl fluoride (PMSF) and 250 mM sucrose; pH 7.5). Homogenates were centrifuged for 30 min at 3220×*g* and 4 ºC, and supernatants stored at − 80 ºC for further analysis. The assays of several enzymes involved in glycogenolysis (GPase [i.GPtotal and ii.GPactive]: glycogen phosphorylase, EC 2.4.1.1), glycolysis (HK: hexokinase, EC 2.7.1.1; PK: pyruvate kinase, EC 2.7.1.40; G3PDH: glycerol-3-phosphate dehydrogenase, EC 1.1.1.8), gluconeogenesis (LDH: lactate dehydrogenase, EC 1.1.1.27; FBP: fructose 1,6-bisphosphatase, EC 3.1.3.11), Krebs Cycle (MDH: malate dehydrogenase, EC 1.1.1.37), pentose phosphate pathway (G6PDH: glucose-6-phosphate dehydrogenase, EC. 1.1.1.49), and lipid metabolism (HOAD: 3-hydroxyacyl-CoA dehydrogenase, EC 1.1.1.35) were performed as previously described for *S. aurata* tissues^31–34^. Enzyme activities were determined using a POWERWAVE 340 microplate spectrophotometer using the KCJUNIOR data analysis software for MICROSOFT. Activities were expressed as specific activities per mg of protein in the homogenate (U mg prot^−1^). Proteins were assayed in duplicate, as described above for plasma samples.

### Proximate composition and fatty acids analysis

Proximate analysis (dry matter, ash, and total protein, N × 6.25) of feed and muscle samples were determined according to^35^ procedures. Lipids were extracted following the Folch method^36^ using chloroform/methanol (2:1 v/v) as solvent, and total lipid content was calculated gravimetrically. Fatty acid (FA) profile was determined by gas chromatography following the method described in^37^, by means of a gas chromatograph (HEWLETT PACKARD, 4890 Series II, Hewlett Packard Company, Avondale, PA), using a modification of the direct transesterification method described by^38^ that involves no prior separation of the lipid fraction.

### Statistical analyses

Results are shown as mean ± standard error of the mean (mean ± SEM). After assessing homogeneity of variance and normality, statistical analysis of the data was carried out using one-way analysis of variance followed by the Tukey test. A comparison of triplicate tanks for all parameters was also performed with one-way analysis of variance. The level of significance was set at p < 0.05. All tests were performed using GRAPHPAD PRISM (v.5.0b) software for Macintosh.

## Results

### Growth performance and biometric parameters

**Figure 1:**
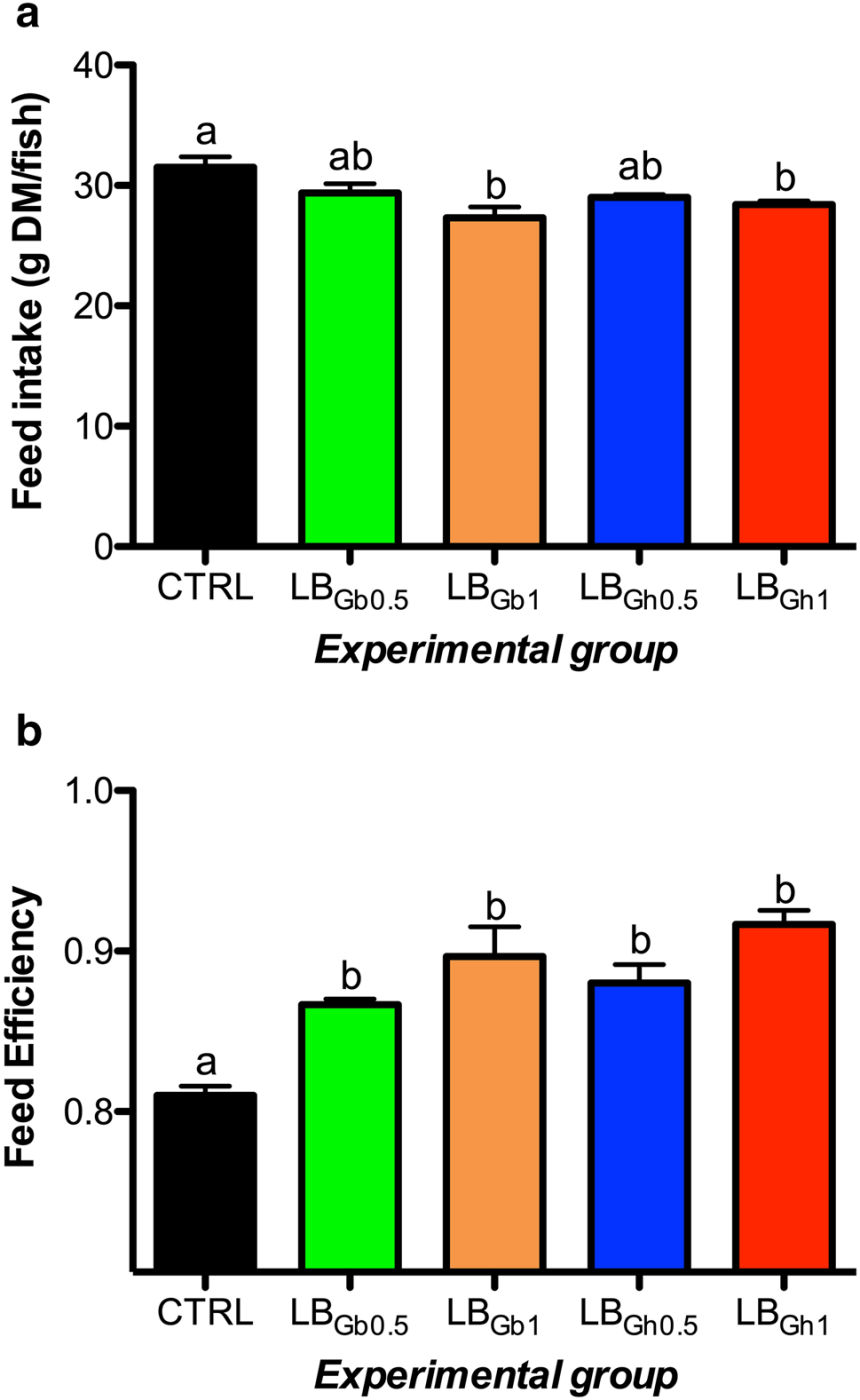
Growth performance related to feed intake (**a**) and feed efficiency **(b)** of gilthead seabream juveniles fed to visual satiety from February to May 2019 (13 weeks) with a control diet and four supplemented diets with 0.5% or 1% of the LB_Gb_ or LB_Gh_ microalgae-derived products. Mean ± SEM values are shown in all panels. Different letters mean statistical differences after one-way ANOVA and Tukey test (p < 0.05). CTRL: control; LB_Gb0.5_: LB-GREENboost (0.5%); LB_Gb1_: LB-GREENboost (1%); LB_Gh0.5_: LB-GUThealth (0.5%); LB_Gh1_: LB-GUThealth (1%).

No mortality occurred during the experiment. In addition, all fish groups grew allometrically from 12–13 g to 37–39 g with an overall weigh gain (WG) of ~ 200% and specific growth rates (SGR) of 1.26–1.30% (Table 3). Diet supplementation significantly reduced feed intake (Fig. 1a) with the subsequent increase of feed efficiency from 0.81 (control group) to 0.87–0.92 in fish fed both compounds (LB_Gb_ and LB_Gh_) and levels of inclusion (0.5 and 1%) (Fig. 1b). Organosomatic indexes calculated as the ratio of tissue to body weight or fork length were determined for liver and intestine. The resulting hepatosomatic index (HSI) was enhanced significantly in fish fed 1% LB_Gb_ diet, whereas the intestine length index (ILI) increased in a dose-dependent manner in fish grown-up with both products (Table 3).

**Table 3:** Growth performance and somatic indexes of juvenile gilthead seabream fed to visual satiety from February to May 2019 (13 weeks) with a control diet and four supplemented diets with 0.5% or 1% of the LB_Gb_ or LB_Gh_ microalgae-derived products.

	CTRL	LB_Gb0.5_	LB _Gb1_	LB_Gh0.5_	LB_Gh1_	*p*^a^
Initial body weight (g)	12.55 ± 0.04	12.64 ± 0.11	12.45 ± 0.10	12.56 ± 0.06	12.54 ± 0.05	0.650
Final body weight (g)	38.12 ± 0.89	37.97 ± 0.86	36.95 ± 1.18	37.89 ± 0.62	39.12 ± 0.36	0.519
Final fork length (cm)	14.08 ± 0.21	14.01 ± 0.25	14.29 ± 0.25	14.15 ± 0.22	14.03 ± 0.21	0.909
CF^b^	1.32 ± 0.05	1.30 ± 0.02	1.30 ± 1.02	1.34 ± 0.04	1.30 ± 0.02	0.835
Weight gain (%)^c^	203.8 ± 6.8	200.5 ± 4.4	196.8 ± 8.3	199.2 ± 5.8	208.1 ± 2.5	0.703
SGR (%)^d^	1.27 ± 0.03	1.26 ± 0.02	1.25 ± 0.03	1.26 ± 0.02	1.30 ± 0.01	0.610
HSI (%)^e^	1.01 ± 0.05^a^	1.16 ± 0.04^ab^	1.24 ± 0.04^b^	1.10 ± 0.07^a^	1.11 ± 0.04^a^	0.037
ILI (%)^f^	96.58 ± 5.09^a^	110.3 ± 5.09^ab^	128.4 ± 8.55^b^	111.1 ± 8.55^ab^	134.5 ± 6.92^b^	< 0.001

Data on body weight, feed intake and growth indexes are the mean ± SEM of triplicate tanks. Data on somatic indexes are the mean ± SEM of 24 fish. Different superscript letters in each row indicate significant differences among dietary treatments based on one-way ANOVA and Tukey’s test (p < 0.05). CTRL: control; LB_Gb0.5_: LB-GREENboost (0.5%); LB_Gb1_: LB-GREENboost (1%); LB_Gh0.5_: LB-GUThealth (0.5%); LB_Gh1_: LB-GUThealth (1%).

^a^Values resulting from one-way analysis of variance.

^b^Condition factor = (100 × body weight)/fork length^3^.

^c^Weight gain (%) = (100 × (body weigh increase)/initial body weight.

^d^Specific growth rate = 100 × (ln final body weight − ln initial body weight)/days.

^e^Hepatosomatic index = (100 × liver weight)/fish weight.

^f^Intestine length index = (100 × intestine length)/fork length.

### Blood and tissue biochemistry

Plasma cortisol levels decreased significantly in fish fed both doses of LB_Gb_ compound, whereas a clear trend, with a ~ 30% of reduction in this hormone, was also observed in both LB_Gh_ groups (Table 4). Dietary supplementation did not alter plasma levels of glucose and proteins, whereas a lowering effect on plasma lactate was found in 0.5% and 1% LB_Gb_ groups and in 1% LB_Gh_ group (Table 4). Moreover, plasma triglycerides significantly increased in a dose-dependent way in fish fed both compounds (LB_Gb_ and LB_Gh_) (Table 4). In the liver, no effects of dietary supplementation were found on the content of triglycerides and glucose (Table 4). However, a significant enhancement in glycogen reserves was detected in fish ingesting the highest dose of both supplements (1% LB_Gb_ and 1% LB_Gh_) (Table 4). In the white skeletal muscle, only fish fed the 1% LB_Gh_ diet experienced a significant (twofold) increase in triglyceride accumulation, whereas the content of glucose, glycogen, and lactate was not modified by dietary supplementation (Table 4).

**Table 4:** Blood and tissue biochemistry of juvenile gilthead sea breams fed to visual satiety from February to May 2019 (13 weeks) with a control diet and four supplemented diets with 0.5% or 1% of the LB_Gb_ or LB_Gh_ microalgae-derived products.

	CTRL	LB_Gb0.5_	LB_Gb1_	LB_Gh0.5_	LB_Gh1_	*p*^a^
Plasma glucose (mM)	3.32 ± 0.08	3.36 ± 0.10	3.59 ± 0.12	3.42 ± 0.11	3.34 ± 0.10	0.385
Plasma lactate (mM)	4.18 ± 0.49^a^	2.26 ± 0.21^b^	2.39 ± 0.31^b^	2.79 ± 0.34^ab^	1.91 ± 0.18^b^	0.012
Plasma triglycerides (mM)	1.25 ± 0.08^a^	1.49 ± 0.17^ab^	1.83 ± 0.14^b^	1.52 ± 0.18^ab^	1.90 ± 0.15^b^	0.032
Plasma proteins (mg mL^−1^)	31.54 ± 0.74	31.84 ± 0.88	30.68 ± 1.64	31.14 ± 1.07	29.44 ± 1.58	0.869
Plasma cortisol (ng mL^−1^)	24.93 ± 3.01^a^	14.51 ± 1.71^b^	14.39 ± 1.89^b^	16.78 ± 2.70^ab^	17.75 ± 1.87^ab^	0.033
Hepatic glucose (µmol gww^−1^)	0.67 ± 0.09	0.82 ± 0.11	0.62 ± 0.14	1.03 ± 0.17	0.67 ± 1.11	0.178
Hepatic glycogen (µmol gww^−1^)	3.86 ± 0.34^a^	4.48 ± 0.23^ab^	5.45 ± 0.39^b^	4.10 ± 0.27^a^	5.23 ± 0.25^b^	0.001
Hepatic triglycerides (µmol gww^−1^)	97.8 ± 11.0	99.7 ± 13.5	104.0 ± 10.3	103.6 ± 8.8	92.6 ± 7.5	0.946
Muscular glucose (µmol gww^−1^)	0.19 ± 0.03	0.19 ± 0.04	0.17 ± 0.03	0.17 ± 0.03	0.19 ± 0.05	0.977
Muscular glycogen (µmol gww^−1^)	0.04 ± 0.02	0.07 ± 0.03	0.09 ± 0.03	0.09 ± 0.04	0.04 ± 0.01	0.136
Muscular lactate (µmol gww^−1^)	41.64 ± 2.57	49.12 ± 2.22	45.22 ± 2.63	47.98 ± 3.32	45.01 ± 2.57	0.330
Muscular triglycerides (µmol gww^−1^)	17.73 ± 2.27^a^	19.84 ± 2.83^a^	18.49 ± 3.07^a^	24.01 ± 4.02^ab^	34.13 ± 5.56^b^	0.022

Data are the mean ± SEM of 12 fish. Different superscript letters in each row indicate significant differences among dietary treatments based on one-way ANOVA and Tukey’s test (p < 0.05). CTRL: control; LB_Gb0.5_: LB-GREENboost (0.5%); LB_Gb1_: LB-GREENboost (1%); LB_Gh0.5_: LB-GUThealth (0.5%); LB_Gh1_: LB-GUThealth (1%).

^a^Values resulting from one-way analysis of variance.

### Metabolic enzymes

**Figure 2:**
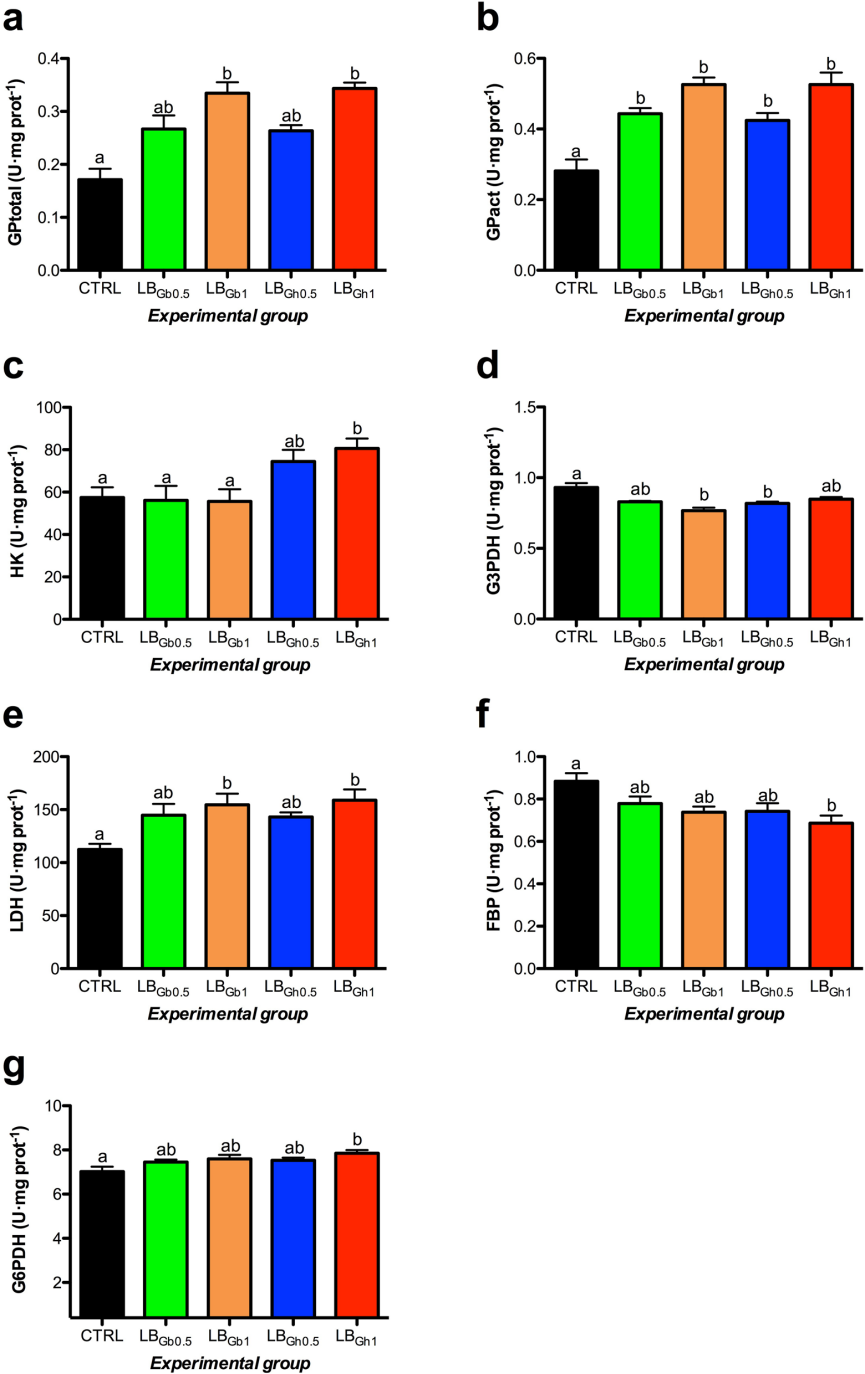
Specific activity (U mg protein^−1^ as mean ± SEM) of metabolic enzymes in the liver of gilthead seabream juveniles fed to visual satiety from February to May 2019 (13 weeks) with a control diet and four supplemented diets with 0.5% or 1% of the LB_Gb_ or LB_Gh_ microalgae-derived products at the end of the feeding trial. Different letters in each panel mean statistical differences after one-way ANOVA and Tukey test (p < 0.05). GPase: glycogen phosphorylase (total: **a**, active: **b**), HK: hexokinase (**c**), G3PDH: glycerol-3-phosphate dehydrogenase (**d**), LDH: lactate dehydrogenase (**e**), FBP: fructose 1,6-bisphosphatase (**f**), G6PDH: glucose-6-phosphate dehydrogenase (**g**). CTRL: control; LB_Gb0.5_: LB-GREENboost (0.5%); LB_Gb1_: LB-GREENboost (1%); LB_Gh0.5_: LB-GUThealth (0.5%); LB_Gh1_: LB-GUThealth (1%).

**Figure 3:**
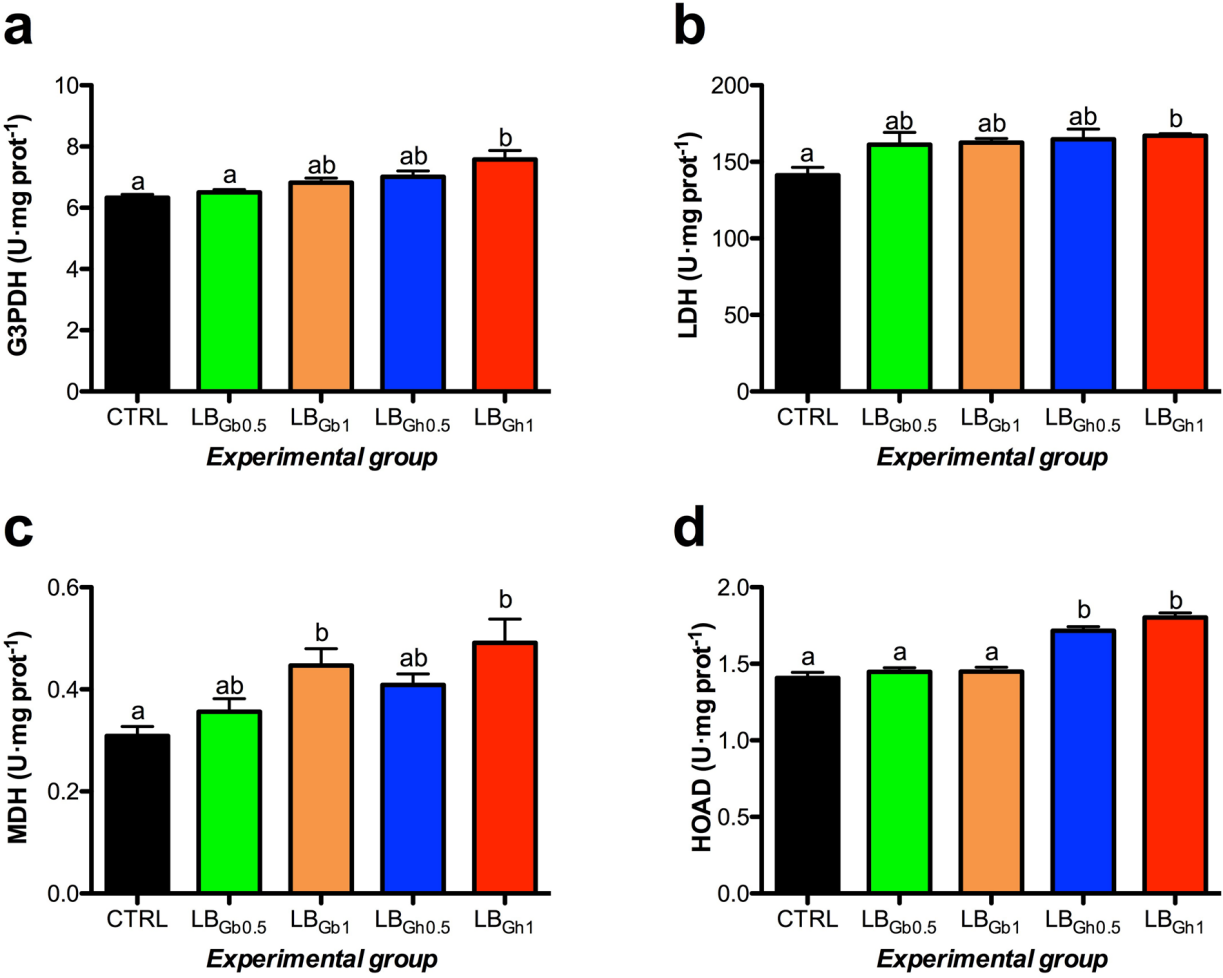
Specific activity (U mg^−1^ protein as mean ± SEM) of metabolic enzymes in the white skeletal muscle of gilthead seabream juveniles fed to visual satiety from February to May 2019 (13 weeks) with a control diet and four supplemented diets with 0.5% or 1% of the LB_Gb_ or LB_Gh_ microalgae-derived products at the end of the feeding trial. Different letters in each panel mean statistical differences after one-way ANOVA and Tukey test (p < 0.05). G3PDH: glycerol-3-phosphate dehydrogenase (**a**), LDH: lactate dehydrogenase (**b**), MDH: malate dehydrogenase (**c**), HOAD: 3-hydroxyacyl-CoA dehydrogenase (**d**). CTRL: control; LB_Gb0.5_: LB-GREENboost (0.5%); LB_Gb1_: LB-GREENboost (1%); LB_Gh0.5_: LB-GUThealth (0.5%); LB_Gh1_: LB-GUThealth (1%).

The effect of dietary supplementation was also evaluated, both in the liver and the white skeletal muscle, on the activity of several metabolic enzymes related to glycogenolysis, glycolysis, gluconeogenesis, Krebs cycle, pentose phosphate pathway, and lipid metabolism. In the liver (Fig. 2), GPase displayed a significant increase with the dietary supplementation with both compounds, being more clearly regulated by both doses (0.5 and 1%) in its total (GPtotal) form. Hepatic HK (glycolysis) and G6PDH (pentose phosphate pathway) activities were enhanced significantly in fish fed the 1% LB_Gh_ diet. An overall opposite pattern was detected for the gluconeogenic enzyme FBP, with the lowest activity found in livers of the 1% LB_Gh_ group. Also, we observed a dose-dependent increase in LDH enzyme with both compounds. In contrast to the observations made on HK activity, the glycolytic enzyme G3PDH shown a significant reduction in its hepatic activity in fish fed both 1% LB_Gb_ and 0.5% LB_Gh_ supplemented diets. No effects were found on the hepatic activity of PK (F = 1.096, p = 0.372), MDH (F = 0.864, p = 0.494) and HOAD (F = 0.352, p = 0.841) enzymes. In the white skeletal muscle (Fig. 3), LDH and G3PDH activities were significantly enhanced in fish fed with the highest dose of LB_Gh_, whereas both doses of this compound (0.5% and 1%) were able to increase HOAD activity. Moreover, a dose-dependent increase in muscle MDH activity was detected when both compounds where used in feed. No dietary effects were found on the muscular activity of GPase total (F = 1.840 p = 0.343) and active (F = 0.669 p = 0.618), HK (F = 0.049 p = 0.995), FBP (F = 0.271 p = 0.895) and G6PDH (F = 1.969 p = 0.121) enzymes, whereas PK activity was found to be at undetectable (ND) levels in this tissue.

### Muscle composition

With regard to muscle overall composition, slight, but not significant differences in lipid and ash contents were observed among experimental groups (Table 5). Notably, the inclusion of any additive in the diet yielded higher muscle total protein content compared to control group, no matter the dose considered, although these differences became more significant in 0.5% LB_Gb_ fillets (Table 5).

**Table 5:** Effects of dietary additives on proximate composition and fatty acid profile of juvenile seabream muscle fed to visual satiety from February to May 2019 (13 weeks) with a control diet and four supplemented diets with 0.5% or 1% of the LB_Gb_ or LB_Gh_ microalgae-derived products.

	CTRL	LB_Gb0.5_	LB_Gb1_	LB_Gh0.5_	LB_Gh1_	*p*^a^
**Proximate composition (% dw)**						
Protein	75.89 ± 0.02^a^	79.69 ± 0.27^c^	78.69 ± 0.28^bc^	77.86 ± 0.17^b^	78.91 ± 0.07^bc^	< 0.001
Lipid	23.00 ± 0.27	24.30 ± 1.13	26.50 ± 0.83	22.70 ± 0.73	23.40 ± 0.83	0.074
Ash	5.81 ± 0.18	6.15 ± 0.18	6.06 ± 0.11	6.25 ± 0.13	5.95 ± 0.33	0.098
Moisture	76.00 ± 0.40	75.70 ± 0.37	75.50 ± 0.17	75.00 ± 0.13	74.30 ± 0.37	0.321
**Fatty acid composition (% of total FAs)**						
14:0	2.02 ± 0.00^a^	2.05 ± 0.00^ab^	2.08 ± 0.00^b^	2.01 ± 0.01^a^	2.09 ± 0.00^b^	0.004
16:0	14.07 ± 0.00	14.01 ± 0.04	14.34 ± 0.01	14.22 ± 0.06	14.13 ± 0.02	0.119
18:0	4.09 ± 0.00	3.94 ± 0.02	4.06 ± 0.01	4.05 ± 0.01	4.02 ± 0.01	0.089
16:1n7	4.56 ± 0.00^a^	4.61 ± 0.01^ab^	4.65 ± 0.00^ab^	4.54 ± 0.00^a^	4.71 ± 0.01^b^	0.024
18:1n7	2.38 ± 0.020^ab^	2.38 ± 0.00^ab^	2.39 ± 0.00^ab^	2.36 ± 0.00^a^	2.41 ± 0.00^b^	0.013
18:1n9	25.30 ± 0.00^ab^	25.61 ± 0.02^bc^	25.63 ± 0.01^bc^	25.16 ± 0.03^a^	25.84 ± 0.05^c^	0.005
20:1n9	1.01 ± 0.00	1.02 ± 0.00	1.03 ± 0.00	1.02 ± 0.00	1.02 ± 0.00	0.309
16:2n4	0.48 ± 0.00	0.48 ± 0.01	0.48 ± 0.01	0.47 ± 0.00	0.46 ± 0.00	0.317
18:2n6	21.00 ± 0.00	20.74 ± 0.03	20.73 ± 0.03	20.82 ± 0.01	20.57 ± 0.15	0.453
18:3n3	1.16 ± 0.00^a^	1.43 ± 0.00^b^	1.74 ± 0.02^c^	1.44 ± 0.02^b^	1.45 ± 0.00^b^	< 0.001
16:3n4	0.47 ± 0.00	0.48 ± 0.00	0.47 ± 0.00	0.47 ± 0.00	0.47 ± 0.00	0.283
18:4n3	0.49 ± 0.00	0.49 ± 0.00	0.47 ± 0.00	0.46 ± 0.00	0.53 ± 0.03	0.410
20:4n6	1.04 ± 0.00	1.01 ± 0.01	1.04 ± 0.00	1.04 ± 0.01	0.99 ± 0.01	0.229
20:4n3	0.45 ± 0.00	0.43 ± 0.00	0.43 ± 0.01	0.44 ± 0.01	0.43 ± 0.00	0.762
20:5n3, EPA	3.30 ± 0.00	3.29 ± 0.01	3.32 ± 0.00	3.31 ± 0.01	3.29 ± 0.01	0.339
22:5n3	1.51 ± 0.00	1.49 ± 0.00	1.49 ± 0.00	1.51 ± 0.01	1.51 ± 0.00	0.218
22:6n3, DHA	12.05 ± 0.01	11.88 ± 0.03	12.06 ± 0.01	12.25 ± 0.11	12.10 ± 0.04	0.383
∑SFA	20.18 ± 0.01	20.00 ± 0.06	20.48 ± 0.02	20.28 ± 0.07	20.23 ± 0.03	0.127
∑MUFA	33.26 ± 0.02^ab^	33.63 ± 0.03^b^	33.70 ± 0.042^bc^	33.09 ± 0.03^a^	33.98 ± 0.07^c^	0.007
∑PUFA	40.99 ± 0.01	40.76 ± 0.01	41.29 ± 0.04	41.27 ± 0.17	40.86 ± 0.10	0.321
Other FA	4.59 ± 0.04	4.36 ± 0.06^a^	3.67 ± 0.01	4.42 ± 0.27	4.30 ± 0.06	0.226
∑n-3	18.95 ± 0.01	19.01 ± 0.03	19.51 ± 0.01	19.41 ± 0.15	19.30 ± 0.06	0.184
∑n-6	22.04 ± 0.00	21.75 ± 0.02	21.78 ± 0.03	21.86 ± 0.02	19.30 ± 0.06	0.416
∑n-9	26.32 ± 0.01^ab^	26.63 ± 0.02^bc^	26.66 ± 0.01^bc^	26.18 ± 0.03^a^	21.56 ± 0.16	0.006
n3/n6	0.86 ± 0.02	0.87 ± 0.00	0.90 ± 0.00	0.89 ± 0.01	0.90 ± 0.01	0.100
EPA/DHA	0.27 ± 0.00	0.28 ± 0.00	0.28 ± 0.00	0.27 ± 0.00	0.27 ± 0.00	0.276

Data are the mean ± SEM of 9 fish. Different superscript letters in each row indicate significant differences among dietary treatments after one-way ANOVA and Tukey’s test (p < 0.05). CTRL: control; LB_Gb0.5_: LB-GREENboost (0.5%); LB_Gb1_: LB-GREENboost (1%); LB_Gh0.5_: LB-GUThealth (0.5%); LB_Gh1_: LB-GUThealth (1%).

^a^Values resulting from one-way analysis of variance.

PUFAs were the prevailing fatty acids in fish muscle at the end of the feeding trial, irrespectively of the diet considered (40- 41% of total FAs), followed by monounsaturated fatty acids (MUFAs, 33%), and then saturated fatty acids (SFAs, 20%) (Table 5). No major differences attributable to experimental diets tested were observed in fatty acid profiles (Table 5). However, although no differences were found in PUFA content, ALA increased in the muscle of all supplement-fed fish, and particularly with LB_Gb_ at 1% (Table 5). The main overall effect of additive inclusion on muscle lipids can be summarized as that MUFA content increased significantly in 1% LB_Gh_, in correspondence with increased oleic acid content in this treatment (Table 5).

## Discussion

Most widely application of microalgae in fish aquaculture is their use, alive or freeze-dried, to enrich zooplankton (i.e. rotifers and *Artemia*), or directly in larval culture tanks for keeping the nutritional quality of the zooplankton used for feeding fish^39,40^. Also, because of their high content of proteins and lipids^7,8^, microalgae have been evaluated in different fish species as alternatives for FM and FO. High production cost of microalgal biomass remains the major drawback of this strategy^20^, although different products (e.g. AlgaPrime DHA, Veramaris), produced at large-scale through fermentation-based technologies, are already being used commercially in salmon feeds^18^. However, there is also an increasing interest in the use of microalgae as nutraceuticals in functional feeds^20,23^, which effects can be exerted at low dietary inclusion level. The products evaluated in this work were included into aquafeeds at levels that can be considered low (1%) or very low (0.5%). Results showed that even at the lowest inclusion level, these products produce positive changes in feed efficiency, welfare and metabolism of gilthead seabream juveniles.

Both products, LB_Gb_ and LB_Gh_, did not affect negatively the specific growth rate of gilthead seabream, the body weight attained by the fish, nor the survival during the trial. Likewise, a combination of microalgae in microdiets for larvae of the same species did not negatively affect growth and survival^17^. Interestingly, both products reduced the feed intake and therefore increased feed efficiency. The combined analysis of growth performance (7.4–13.5% improvement in feed efficiency) indicates that the incorporation of these products in functional feeds for the gilthead seabream would reduce in up to 148 kg the amount of feed needed to produce one ton of farmed fish, and in saving up to 222 € per ton of fish produced, with a different balance in terms of economic issues depending on the inclusion level (Table 6), but always producing positive effects related to welfare and metabolic status (see below). From our results, it is apparent that these effects were achieved through common and differential actions of the products on fish physiology, likely as a result of their composition. While both products increased the length of the intestine and presumably its absorptive capacity, the higher values were observed for 1% LB_Gh_. As in other marine fish species^41^, the inclusion of different microalgae in fry^42^ and juveniles^43^ of the gilthead sea bream increases enterocyte absorption surface. The same effect has been observed through dietary supplementation with *Bacillus*-based probiotics^44^. Intestine length, on the other hand, is known to be also a plastic trait as shown before in this fish species in response to plant proteins^5,45^ but, to the best of our knowledge, not reported before to respond to low or very low levels of microalgae-derived nutraceuticals such as in this work. Conversely, LB_Gb_ was the only product that changed the hepatosomatic index and cortisol level in plasma, exerting this effect even at 0.5% inclusion in feed, and supporting that LB_Gb_ and LB_Gh_ exert different effects on fish metabolism.

**Table 6:** Estimation of using microalgae-based products on the feed saving and global balance of feed costs for producing one ton of farmed fish.

	Feed efficiency (kg fish kg feed^−1^)	Total feed (kg feed ton fish^−1^)	Feed saving (kg feed ton fish^−1^)	Cost feed saving (€ ton fish^−1^)^a^	Additive use (kg ton fish^−1^)	Additive cost (€ ton fish^−1^)	Balance (€ ton fish^−1^)^b^
CTRL	0.81	1235					
LB_Gb0.5_	0.87	1149	85	128	5.75	114.9	13.1
LB_Gb1_	0.90	1111	123	185	11.11	222.2	− 37.2
LB_Gh0.5_	0.88	1136	98	147	5.68	113.6	33.4
LB_Gh1_	0.92	1087	148	222	10.87	217,4	4.6

CTRL: control; LB_Gb0.5_: LB-GREENboost (0.5%); LB_Gb1_: LB-GREENboost (1%); LB_Gh0.5_: LB-GUThealth (0.5%); LB_Gh1_: LB-GUThealth (1%).

^a^For the estimation it has been considered a value of 1.5€ kg feed^−1^.

^b^Estimated as *cost feed saving* (€ ton fish^−1^) – *additive cost* (€ ton fish^−1^).

Under stressful conditions, cortisol plays a key regulatory role in skeletal muscle metabolism, inhibiting glycogen synthesis^46^, and inducing the mobilization of glucose and fatty acids to overcome the stress stimulus^47^. In the liver, cortisol also induces energy substrate repartitioning to cope with the enhanced energy demand associated with stressor exposure^48^. However, the roles of basal level of cortisol in fish under non-stressed conditions remain not completed understood^49^. In this regard, chronic oral administration of cortisol to gilthead seabream leads to higher energy expenditure and lower growth rate, increased hepatic triglycerides content and enhanced amino acid catabolism and gluconeogenesis in muscle^49^. Conversely, low level of cortisol may stimulate GH-induced IGF-1 expression in fish hepatocytes^50^, protein synthesis (i.e. somatic growth), and deposition of hepatic glycogen and lipid in the muscle of fish^49,51,52^. These effects may be exerted by supplementation with microalgae-based products in our study, as suggested by the higher feed efficiency, higher hepatic glycogen content, and higher triglyceride levels in muscle of 1% LB_Gb_ and 1% LB_Gh_ fed fish. It is known that dietary fatty acids play important roles in the regulation of cortisol release in fish. For instance, in gilthead seabream, dietary deficiencies on n-3 HUFA raised the basal plasma cortisol levels and altered the pattern of cortisol release after stress^53^, and high concentration of arachidonic acid (ARA) and eicosapentaenoic acid (EPA) reduced cortisol secretion in this species^54,55^. However, given the low inclusion level of LB_Gb_ in this study, it may exert its effects on cortisol levels through a different mechanism, that remains unknown, although it can be suggested that the lower doses of aquafeeds needed to accomplish apparent satiety produces a decrease in anticipatory activity that leads to improve the welfare status of the fish, which is clearly reflected in lower cortisol levels. Even so, whether lower levels of baseline cortisol in plasma also affect the response of fish to stressful condition such as high stocking density or handling remains unknown and deserves further investigation.

To further study the biochemical basis of the observed phenotypic outputs of microalgae-based product supplementation, and to better differentiate the metabolic effects of LB_Gb_ and LB_Gh_, we measured the level of different metabolites, and the activity of several metabolic enzymes, both in the liver and white skeletal muscle. We observed that both products lowered plasma lactate levels, although LB_Gb_ promoted this effect at a lower inclusion (0.5%) than LB_Gh_ (1%). This result suggests that both products may favor oxidative over anaerobic metabolism in the white skeletal muscle, or that lactate uptake and clearance by the liver or other tissues is stimulated. We found no differences among treatments in glycogen, free glucose, and lactate in the white skeletal muscle, and the activity of LDH in muscle of all supplemented fed fish was not different to that of control fish, except for slightly higher values in 1% LB_Gh_. Thus, our observations do not sustain a lower production of lactate from muscle anaerobic metabolism in fish fed diets supplemented with the microalgae-based products. Interestingly, the higher hepatic storage of glycogen in fish fed diets supplemented with LB_Gh_ and LB_Gb_ at 1% and a trend for hepatic LDH activity to increase suggests that both products may promote the hepatic uptake of lactate. However, the conversion of lactate to pyruvate by LDH in the liver (i.e. Cori cycle), and its further conversion to glycogen, is not clear in fish. Indeed, an early study analyzing lactate metabolism in 36 fish species suggested that little blood lactate is taken up by the liver in fish^56^, and more recent studies also suggested that using lactate as a precursor for liver glycogen is unlikely in fish^57^. Other tissues of gilthead seabream using lactate as energy source^58,59^ should also contribute to the clearance of lactate^57,60^ and part of the lactate could return to the muscle^60^. Our observations on plasma lactate would be also related with a decrease in the level of fish activity when fed the micro-algae supplemented diets, and this may in turn be associated with decreased cortisol levels and improved feed efficiency as discussed above.

We also found that the inclusion of LB_Gh_ increased hepatic HK activity, most significantly at 1% inclusion in feed, while PK activity was unaltered by dietary supplementation. HK is the first step in glycolysis, phosphorylating glucose to be used by cells, while PK catalyzes the last step of glycolysis producing pyruvate and ATP. Together, these results support that 1% LB_Gh_ supplementation enhanced the liver capacity for glucose uptake, which seems to be stored as glycogen instead of being oxidized for energy. This inferred scenario agrees with reduced hepatic gluconeogenic enzyme (FBP) activity in 1% LB_Gh_ fed fish. A non-significant trend for FBP to decrease activity in 1% LB_Gb_ fed fish may explain why this fish also exhibited more hepatic glycogen than control fish. However, it remains unknown the metabolic significance of higher activities of GP in 1% LB_Gh_ and 1% LB_Gb_ groups. It would be related with the turnover of liver glycogen for glucose to be used in other metabolic pathways such as the synthesis of fatty acids. Increased glucose uptake by the liver, or production of glucose from glycogen, is known to have a stimulatory effect on the lipogenic enzymes G6PDH and MDH, which provide NADPH for the biosynthesis of fatty acids, and that this leads to a higher lipid storage or export form the liver^61,62^. Indeed, we found for hepatic G6PDH a trend to increase its activity with microalgae supplementation, with higher activity for 1% LB_Gh_, while MDH exhibited higher values (although non-significant) in both LB_Gh_ and LB_Gb_ at 1% inclusion. The absence of differences in hepatic triglycerides stored in our study supports the export as the most likely fate of synthesized triglycerides, in agreement with a higher triglyceride level in plasma in 1% LB_Gh_ and 1% LB_Gb_ fed fish. This would be also the cause of increased triglyceride content in muscle of 1% LB_Gh_ groups, doubling the triglyceride content of muscles of control fish. It is difficult to fully explain the effect of 1% LB_Gh_ on muscle triglycerides, as fat deposition depends upon balance between various metabolic pathways and trade-offs among different organs. Some of our observations suggest that de novo lipogenesis (DNL) in the muscle might contribute to higher triglyceride content in 1% LB_Gh_ fed fish. For instance, we observed higher activity for the lipogenic enzyme MDH in muscle in 1% LB_Gh_ and 1% LB_Gb_, with the highest mean value induced by the former. MDH activity has been related to intramuscular fat content in other meat-producing animals^63^. However, the contribution of DNL in muscle to fat accumulation is debatable in fish as it mostly takes place in the liver^64^. Indeed, we found no evidence of differences in muscle DNL among treatments by using the DNL index (16:0/18:2n6 ratio = 0.7). Higher activity of G3PDH in 1% LB_Gh_ fed fish is also in agreement with higher triglyceride content of muscle, as it is a marker of lipid synthesis in fish^65^ and other animals^63^. This enzyme produces glycerol-3-phosphate to which fatty acids are esterified, thus it is involved in the synthesis of triglycerides from imported fatty acids (e.g. from liver) rather than in muscle DNL. Finally, we recognize that changes in the triglyceride content of muscle of 1% LB_Gh_ fed fish may also result from modifications in the metabolic fate of other nutrients induced by this microalgal product. For instance, in gilthead seabream, between 22 and 30% of the total lipid deposited may come from dietary proteins^66^.

One interestingly finding of this study is the higher muscular HOAD activity, the third step of beta oxidation, in fish with increased muscle triglycerides (i.e. 1% LB_Gh_ fed fish). It is known that in higher vertebrates, lipid availability per se increases mitochondrial fatty acid oxidative capacity in muscle^67^. In fish (rainbow trout), an experimental high muscle fat line is known to exhibit an enhanced fatty acid oxidation potential^68^. Increased HOAD activity in our study may be a compensatory mechanism to control excessive fat accumulation in fish muscles supplemented with 1% LB_Gh_, or might be involved in lipid remodeling within the muscle.

The total content of lipids and n-3 PUFAs did not vary across groups. This result was somewhat expected as experimental diet were rich in FM and FO. Yet, we observed a non-significant trend for n-3 PUFAs to increase with supplementation, probably derived from higher ALA (18:3n-3) content in all supplemented groups. We have no explanation for this increase in ALA content, but its effects might be related with the observed high protein content in muscle of microalgae fed fish, as ALA is known to prevent muscle wasting in higher vertebrates^69^. We found, on the other hand, that supplementation with LB_Gb_ both at 0.5% and 1%, and with LB_Gh_ at 1%, resulted in fish muscles with higher MUFA content. This observation is in agreement with the evidenced increase in oleic acid content, which is the main MUFA of muscle in gilthead sea bream and other fish species (~ 70% of total MUFAs in rainbow trout^70^). This result is also in accordance with the boost in muscle triglycerides in 1% LB_Gh_ fed fish, as triglycerides are rich in MUFA. Therefore, it seems that storage lipids became slightly enriched in MUFA after feeding the microalgae products. Enhanced MUFA in fish muscle is thought to be positive, as long as SFA and n-6 PUFA do not increase, and the n-3 to n-6 ratio is not altered, as occurred in our study. Given that our assessment was performed in juveniles, major effects of increased MUFA and the ALA (n-3 PUFA) in muscle should be related with physiological processes (e.g. permeability and fluidity of membranes) and overall health status. In rats, dietary ALA supplementation increased the movement of lipids across the sarcolemmal membrane, a rate-limiting step in fatty acid oxidation, and led to higher triglyceride content and rates of fat oxidation^71^, while it is known to have antioxidant and anti-inflammatory effects in both rats and humans^72^. However, the use of the products evaluated in this study in finishing diets for adult gilthead sea bream farmed on diets with high substitution of FM and FO is worthy to be also explored. Information on the use of dietary supplements to increase the efficiency of finishing protocols is scarce, and their effects on muscle fatty acid composition would be different in fast growing juveniles (this study) and in commercial size (near harvest) fish. Moreover, given that in this and other fish species the content of lipids in fillet is correlated with the levels of different LC-PUFA^73^, and that 1% LB_Gh_ increases the triglyceride content of muscle, it would result in adults with more MUFAs, and maybe PUFAs, per gram of fillet. Although dietary FA profiles are generally reflected in fish muscle^74–76^, the significant increase in muscle MUFAs observed in the present study could be attributed to slightly differences in dietary fatty acids as a result of additive inclusion. The marginal higher content of 18:1n-9 and 18:3n-3 in the dietary treatments supplemented with the microalgae products might contribute to the observed increase of those fatty acids in the muscle of fish compared to the results obtained for the control diet (Table 2).

In general, this study demonstrated that the use of LB_Gb_ and LB_Gh_ additives in gilthead seabream diets does not affect growth and improve feed efficiency. Different positive effects of microalgae products in feeds have been achieved at low level of supplementation such as 5% in salmon^77^, 5–7% in pigs^78^, and only 0.1–0.2% in broiler chicks^29^. In our study, it was found that LB_Gb_ and LB_Gh_ exerted their positive, although different, effects at 0.5–1% in gilthead seabream juveniles. Given this low inclusion level in diet (e.g. 1%) and their reasonable cost (about 20€ per kg), the use of these products would result in saving from 85 to 148 kg of feeds per ton of farmed fish, which might reduce feed cost up to 33.4 € per ton of fish in the case of LB_Gh_ (Table 6). Moreover, the inclusion of these microalgae-based products resulted in functional feeds that, depending on the product type and its inclusion level, produce low plasma cortisol level, higher hepatic glycogen content, and higher triglycerides, ALA and MUFA levels in muscle. Further studies are needed to elucidate if these benefits observed under controlled conditions can be extended to different commercial species, challenging culture conditions (e.g. high stocking densities, handling, or metabolic depletion due to overwintering), or in improvements in fillet quality after long-term feeding. Similarly, it would be worthy to test the potential of these products to ameliorate some of the negative effect of high FM and FO replacement in gilthead seabream feeds, especially those targeting the intestine and lipid metabolism.

## Data Availability

All data generated or analyzed during this study are included in this published article.
